# Impact of Frailty on Prognosis and Functional Outcome in Patients With Acute Respiratory Deterioration of Interstitial Lung Disease

**DOI:** 10.1155/pm/3510542

**Published:** 2026-04-06

**Authors:** Yuta Takahashi, Mitsutoshi Akiho, Takuya Miyahara, Yasunari Sakai, Shuken Kobayashi, Daisuke Minamishima, Shuichi Nakada, Kanji Yamada, AIR REHAB-ILD Study Collaborators

**Affiliations:** ^1^ Department of Rehabilitation, St. Luke′s International Hospital, Tokyo, Japan, luke.ac.jp; ^2^ Department of Rehabilitation Sciences, University of Tokyo Health Sciences, Tokyo, Japan; ^3^ Department of Rehabilitation, Mitsui Memorial Hospital, Tokyo, Japan, mitsuihosp.or.jp; ^4^ Department of Rehabilitation, Shinshu University Hospital, Nagano, Japan, shinshu-u.ac.jp; ^5^ Department of Rehabilitation, Kitasato University Hospital, Kanagawa, Japan, kitasato-u.ac.jp; ^6^ Department of Rehabilitation, Tokyo Metropolitan Police Hospital, Tokyo, Japan, keisatsubyoin.or.jp; ^7^ Department of Rehabilitation, St. Marianna University School of Medicine Hospital, Kanagawa, Japan; ^8^ Department of Rehabilitation, Kobe City Medical Center General Hospital, Hyogo, Japan, chuo.kcho.jp

**Keywords:** frailty, hospitalization, interstitial lung disease, mortality, pulmonary rehabilitation

## Abstract

**Background:**

Frailty has emerged as an important prognostic factor in patients with interstitial lung disease (ILD), but its impact on hospitalized ILD patients with acute respiratory deterioration (ARD) remains unclear. The objective of this study is to investigate the association between preadmission frailty and clinical outcomes including 180‐day mortality and functional decline in ILD patients hospitalized due to ARD.

**Methods:**

This multicenter prospective study included ILD patients admitted due to ARD at five acute care hospitals in Japan. Frailty was assessed at admission using the Clinical Frailty Scale (CFS) based on patients′ prehospitalization condition. The primary outcome was 180‐day mortality from the day of admission. Secondary outcomes included early ambulation and functional decline at discharge. Cox regression and logistic regression analyses were used to assess the impact of frailty on outcomes.

**Results:**

Among 205 patients, 37 (18.0%) were classified as frail (CFS 5–9). The frail group had significantly higher 180‐day mortality than the nonfrail group (*p* = 0.002). Frailty remained an independent predictor of 180‐day mortality after multivariable adjustment (hazard ratio 2.450, 95% confidence interval [CI]1.327–4.524, *p* = 0.004). Nonfrailty was an independent predictor of early ambulation (odds ratio [OR] 4.820, 95% CI 1.700–13.600, *p* = 0.003); however, it was not a significant predictor of functional decline (OR 1.810, 95% CI 0.748–4.380, *p* = 0.188).

**Conclusions:**

Frailty independently predicted mortality and adverse functional outcomes in ILD patients hospitalized due to ARD. Frailty assessment on admission may help identify patients who require early and tailored multidisciplinary interventions.

## 1. Introduction

Interstitial lung disease (ILD) encompasses a wide variety of diseases that cause inflammation with the alveolar septum as the primary site of involvement.[1] Hospitalization due to acute respiratory deterioration (ARD), including acute exacerbation (AE), is common during the clinical course of ILD.[2] The prognosis of hospitalized ILD patients is extremely poor in both idiopathic pulmonary fibrosis (IPF) and non‐IPF cases, with high short‐ and long‐term mortality rates, and improving outcomes in these patients remains a clinical challenge.[3–5] Previous studies have identified several predictors of in‐hospital mortality, such as male sex, smoking history, a diagnosis of IPF, use of invasive mechanical ventilation, body mass index, and clinical severity score.[4, 5] In addition to these factors, frailty has recently been recognized as a prognostic factor in stable ILD cohorts. [6–9] These previous studies have shown that frailty is associated with an increased risk of mortality, even when adjusted for age, gender, baseline lung function, and ILD diagnosis. Moreover, frailty is potentially considered a modifiable factor in a patient population with few therapeutic options. However, the impact of frailty on mortality in hospitalized patients with ILD is unclear. Although one prior study investigated this association, [10] it was conducted at a lung transplant center and included a high proportion of transplant recipients, limiting interpretation of the direct effect of frailty on prognosis. [11] In addition to its impact on survival, frailty is also expected to influence functional outcomes; however, this association has not been well studied in hospitalized ILD patients. Therefore, this study is aimed at clarifying the impact of preadmission frailty on prognosis and functional outcomes in ILD patients hospitalized due to ARD, without lung transplantation.

## 2. Methods

### 2.1. Study Design

This was a multicenter, prospective observational study conducted at five acute care hospitals in Japan. Patients with ILD who were hospitalized for ARD were enrolled between September 1, 2021, and June 30, 2024. We excluded patients who were nonambulatory prior to admission (i.e., unable to walk even with assistance), those who declined participation, and those with missing key data required for analysis. The diagnosis of ILD was verified by multidisciplinary discussion based on chest high‐resolution computed tomography. ARD was defined as an acute worsening or new onset of dyspnea typically within 1 month, which required hospitalization. [2] AE‐ILD was defined as a subset of ARD characterized by (1) new bilateral ground‐glass opacities or consolidation superimposed on a background of fibrotic lung disease and (2) no identifiable extraparenchymal cause such as pulmonary embolism, pneumothorax, or heart failure, according to the 2016 international working group criteria. All patients received standard in‐hospital pulmonary rehabilitation by physiotherapists. Pulmonary rehabilitation mainly focused on early mobilization at the acute medical ward and the patients′ bedside. Early mobilization, including sitting on the edge of the bed, moving from bed to chair, standing, stepping in place, and walking, was gradually progressed using adequate oxygen and respiratory support device to keep percutaneous arterial oxygen saturation above 88%. The study protocol was approved by the ethics committees of all participating institutions (Ethics Committee of St. Luke′s International Hospital, Approval Number: 21‐R075, date of approval: August 4, 2021) and conducted in accordance with the principles of the Declaration of Helsinki. Written informed consent was obtained from all participants or their surrogates. This study also adhered to the STROBE guidelines.

### 2.2. Data Definition and Collection

Clinical and demographic data were collected, including age, sex, ILD subtype, prior use of long‐term oxygen therapy, laboratory data, and treatments such as mechanical ventilation and steroid pulse therapy.

Frailty was assessed using the Clinical Frailty Scale (CFS), [12] based on the patient′s condition prior to hospitalization. The CFS is a 9‐point scale that assesses a patient′s level of physical frailty from 1 (*very fit*) to 9 (*terminally ill*) based on their functional abilities and need for assistance in daily activities. CFS scores were determined by trained physiotherapists at each institution through interviews with the patient and/or their family members. Following previous studies, [13] patients were classified into two groups: the nonfrail group (CFS 1–4) and the frail group (CFS 5–9).

The primary outcome was 180‐day all‐cause mortality from the date of hospital admission. This included in‐hospital deaths, and patients who remained alive at 180 days or were lost to follow‐up before 180 days were treated as censored observations. As secondary outcomes, we assessed (1) early ambulation, defined as the ability to walk (with or without assistance) within 2 days of admission, and (2) in‐hospital functional decline, defined as a reduction in total Barthel Index (BI) score at discharge compared with the prehospitalization score according to previous studies. [14] The preadmission BI score was obtained through interviews, and the discharge BI score was based on records from physiotherapists.

### 2.3. Statistical Analysis

Continuous variables are expressed as median (interquartile range), and categorical variables are described as numbers (percentages). Group comparisons between the frail and nonfrail groups were performed using the Mann–Whitney *U* test for continuous variables and the chi‐square test or Fisher′s exact test for categorical variables, as appropriate. Kaplan–Meier survival curves were generated to estimate 180‐day mortality from the date of hospital admission, and the log‐rank test was used to compare survival between the frail and nonfrail groups. Univariate and multivariate Cox proportional hazards regression analyses were performed to evaluate the association between frailty and 180‐day mortality. In the multivariate model, adjustment was made for prespecified confounders based on prior studies: age, sex, use of mechanical ventilation, steroid pulse therapy, and diagnosis of IPF. [4, 5] As a sensitivity analysis, we conducted Cox regression with stepwise variable selection based on the Akaike Information Criterion (AIC), including all preadmission variables except frailty, to examine whether frailty retained independent prognostic significance. For secondary outcomes, multivariate logistic regression analyses were performed to assess associations between frailty and (1) early ambulation and (2) in‐hospital functional decline, adjusting for relevant covariates. As this study was conducted as a subanalysis of the AIR‐REHAB ILD study, a study evaluating the safety and efficacy of pulmonary rehabilitation in hospitalized ILD patients, a prespecified power analysis for the current analysis was not performed. Missing data were handled by complete case analysis. All the reported *p* values were two‐sided, and *p* values of < 0.05 were regarded as statistically significant. All statistical analyses were performed using R software (Version 4.4.1; R Foundation for Statistical Computing, Vienna, Austria).

## 3. Results

**Figure 1:**
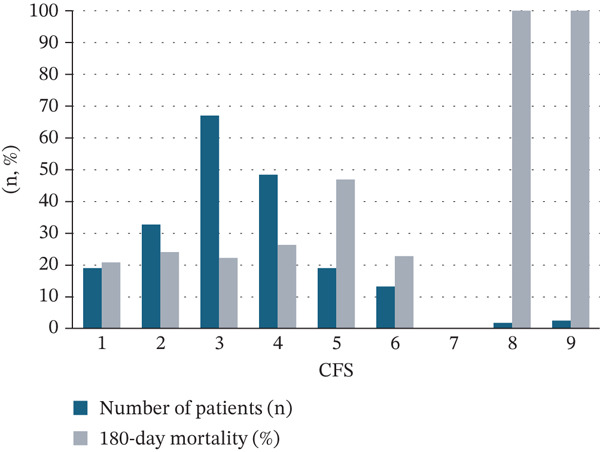
Distribution of patients and 180‐day mortality rate according to the Clinical Frailty Scale.

From a total of 290 patients screened, 205 consecutive patients met the inclusion criteria and were eligible for this study, after excluding 85 patients due to missing CFS data. Thirty‐seven patients (18.0%) were classified as frail (CFS 5–9) and 168 as nonfrail (CFS 1–4). A histogram showing the number of patients and 180‐day mortality rate at each CFS is shown in Figure 1. CFS 3 was the most common with 67 cases (32.7%), whereas two patients (0.9%) had CFS 8 and three patients (1.5%) had CFS 9. The frail group was older (median 79.0 vs. 75.0 years) and had a lower BMI (median 21.1 vs. 22.9 kg/m^2^) compared with the nonfrail group (Table 1). The prevalence of prior long‐term oxygen therapy use, prior ILD diagnosis, and prior ILD exacerbation was also higher in the frail group. In‐hospital mortality was 27.0% in the frail group and 15.5% in the nonfrail group, although the difference was not statistically significant.

**Table 1:** Characteristics and outcomes of study population (*n* = 205).

	Nonfrail (*n* = 168)	Frail (*n* = 37)	*p*
Age, years	75.0 (67.8, 80.0)	79.0 (74.0, 83.0)	0.004
Male, *n* (%)	131 (78.0)	28 (75.7)	0.828
BMI, kg/m^2^	22.9 (20.4, 24.6)	21.1 (19.1, 23.7)	0.034
Ever smoker, *n* (%)	122 (72.6)	28 (75.7)	0.768
mMRC grade, *n* (0/1/2/3/4)	18/34/37/50/20	0/2/2/9/24	< 0.001
%FVC, % (*n* = 85; 17)	73.7 (59.0, 87.6)	71.5 (55.7, 85.4)	0.650
Prior LTOT use, *n* (%)	31 (18.5)	20 (54.1)	< 0.001
Prior ILD diagnosis, *n* (%)	102 (60.7)	31 (83.8)	0.008
Prior ILD exacerbation, *n* (%)	50 (29.8)	22 (59.5)	0.001
Types of ILD			0.577
UIP/IPF, *n* (%)	71 (42.3)	21 (56.8)	
IIPs, *n* (%)	31 (18.5)	9 (24.3)	
CTD‐ILD, *n* (%)	35 (18.2)	5 (13.5)	
Drug induced, environmental, *n* (%)	29 (17.3)	2 (5.4)	
AE‐ILD, *n* (%)	114 (67.9)	15 (40.5)	0.003
Steroid pulse therapy, *n* (%)	103 (61.3)	20 (54.1)	0.461
Mechanical ventilation, *n* (%)	11 (6.5)	2 (5.4)	1.000
High‐flow nasal cannula, *n* (%)	31 (18.5)	12 (32.4)	0.074
WBC, 10^3^/*μ*L	9.6 (6.8, 12.2)	10.6 (8.3, 13.6)	0.111
CRP, mg/dL	5.4 (1.2, 12.6)	5.5 (1.0, 13.2)	0.662
LDH	283.5 (231.2, 385.5)	301.1 (252.0, 394.5)	0.309
KL‐6, U/mL	961.5 (601.0, 1754.5)	954.0 (574.5, 1546.8)	0.758
Time to initial rehabilitation, days	2 (1, 5)	2 (1, 4)	0.496
Time to initial ambulation, days	3.0 (1.0, 6.0)	6.0 (3.3, 9.5)	0.006
Length of hospital stay, days	23.0 (14.0, 38.3)	24.0 (15.0, 37.0)	0.888
In‐hospital mortality, *n* (%)	26 (15.5)	10 (27.0)	0.100
Outcomes			
Early ambulation, *n* (%)	67 (39.9)	5 (13.5)	0.002
Functional decline, *n* (%)	75 (44.6)	24 (64.9)	0.030
180‐day mortality, *n* (%)	40 (23.8)	17 (45.9)	0.007

*Note:* Values are expressed as median (interquartile range) or *n* (%).

Abbreviations: AE‐ILD, acute exacerbation of interstitial lung diseases; BMI, body mass index; CRP, C‐reactive protein; CTD‐ILD, connective tissue disease‐related interstitial lung diseases; FVC, forced vital capacity; IIPs, idiopathic interstitial pneumonias; ILD, interstitial lung diseases; KL‐6, sialylated carbohydrate antigen KL‐6; LDH, lactate dehydrogenase; LTOT, long term oxygen therapy; mMRC, modified medical research council; UIP/IPF, usual interstitial pneumonia/idiopathic pulmonary fibrosis; WBC, white blood cell.

**Figure 2:**
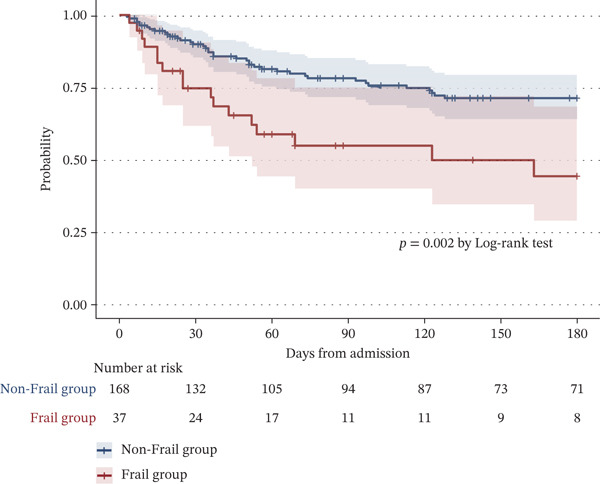
Kaplan–Meier survival curves for 180‐day mortality according to frailty status. The *y*‐axis (vertical axis) shows survival rates, and the *x*‐axis (horizontal axis) shows the observation period from the date of hospitalization. The *p* value is the result of the log‐rank test.

A total of 57 deaths occurred during the observation period, which had a median duration of 98 days (interquartile range: 33–180). The Kaplan–Meier analysis demonstrated significantly higher 180‐day mortality in the frail group compared with the nonfrail group (*p* = 0.002; Figure 2). In univariate Cox regression analysis, frailty was significantly associated with increased 180‐day mortality (hazard ratio [HR] 2.404, 95% confidence interval [CI] 1.360–4.250, *p* = 0.003), and this association remained significant in multivariate analysis (HR 2.450, 95% CI 1.327–4.524, *p* = 0.004) (Table 2). In a sensitivity analysis using variable selection based on the AIC, frailty (HR 2.474, 95% CI 1.268–4.825, *p* = 0.008), steroid pulse therapy, and prior long‐term oxygen therapy use were also independently associated with 180‐day mortality (Table S1). Nonfrailty was significantly associated with early ambulation in both univariate and multivariate logistic regression analyses (odds ratio [OR] 4.820, 95% CI 1.700–13.600, *p* = 0.003) (Table 2). However, frailty was not significantly associated with functional decline (OR 1.810, 95% CI 0.748–4.380, *p* = 0.188) (Table 2).

**Table 2:** Prognostic impact of frailty on outcomes.

	Univariable		Multivariable	
	HR (95% CI)	*p*	HR (95% CI)	*p*
180‐day mortality	2.404 (1.360, 4.250)	0.003	2.450 (1.327, 4.524)	0.004
	OR (95% CI)	*p*	OR (95% CI)	*p*
Early ambulation	4.250 (1.570, 11.400)	0.004	4.820 (1.700, 13.600)	0.003
Functional decline	2.230 (0.970, 5.130)	0.059	1.810 (0.748, 4.380)	0.188

*Note:* Adjusted values for age, sex, use of mechanical ventilation, steroid pulse therapy, and diagnosis of IPF are shown in the multivariate analysis.

Abbreviations: CI, confidence interval; HR, hazard ratio; OR, odds ratio; UIP/IPF, usual interstitial pneumonia/idiopathic pulmonary fibrosis.

## 4. Discussion

This Japanese multicenter prospective study demonstrated that frailty is associated with poor prognosis in ILD patients hospitalized for ARD. The observed association extends prior evidence from stable ILD cohorts, [3, 7, 8, 15–17] reinforcing the clinical importance of assessing frailty during hospitalization. Frailty may also hinder early ambulation and contribute to subsequent functional decline.

### 4.1. Comparison With Previous Studies

Unlike many previous studies that focused on stable outpatients, [3, 7, 8, 15–17] this study targeted hospitalized ILD patients. A prior study by Hollebeke et al. reported no association between frailty and 1‐year mortality after AE‐ILD when adjusting for lung transplantation as a competing risk. [10] In that cohort, nearly half of the patients received lung transplants after discharge, which may have offset the impact of frailty. In contrast, our study focused on the natural course of hospitalized patients without transplant, providing new evidence on the prognostic significance of frailty in this setting. At the same time, it should be considered that the inclusion of patients with CFS 8–9, representing the most severe frailty, may have influenced the observed outcomes in this study. Furthermore, this is, to our knowledge, the first report to explore the relationship between frailty and early ambulation or in‐hospital functional decline in ILD patients. Early ambulation may prevent functional decline during hospitalization, [18] but few patients achieved early ambulation, about 40% even in the nonfrail group and 10% in the frail group. As a result, approximately 40% of the nonfrail group and more than 60% of the frail group had functional decline compared with prehospitalization. The incidence of functional decline in ILD patients is high, since a previous study that reported the incidence of functional decline in elderly hospitalized patients using a similar definition found that approximately 30% of patients developed functional decline. [14] In this study, frailty did not emerge as a significant variable as a risk factor for functional decline; however, these associations warrant further investigation using various assessment scales and definitions.

In this study, we used ARD not AE‐ILD as our include criteria. The reason is that most patients with ILD are known to have respiratory failure that is not a cause of hospitalization for AEs, and the prognosis for these patients is poor. [19–21] Therefore, this study is intended for a more general population of patients in clinical practice. As a result, mortality in this study was lower than in previous AE‐ILD studies, [2] which may have indirectly strengthened the association between frailty and 180‐day mortality in this study. The impact of frailty is likely to depend on whether the patient has AE‐ILD or not, because frailty tends to have less prognostic impact when the severity of the disease is higher.

The 18% prevalence of frail in the present study was lower than in previous studies (26%–55%) [8, 10, 15–17] but similar to previous studies (21%) using the CFS as in our study. [7] The CFS is simple, easy to incorporate into clinical settings, and has been shown to be associated with prognosis in a variety of disease groups and hospital and community settings. [13, 22–24] Meanwhile, the Fried frailty phenotype assessment and Frailty index used in previous studies in stable ILD patients include multiple factors such as nutritional status, comorbidities, and laboratory data, in addition to physical activity, increasing the probability of being classified as frail. [8, 10, 15–17] Therefore, it is not appropriate to directly compare the prevalence of frailty assessed by different scales, and it is important that both scales are related to clinical outcomes.

### 4.2. Mechanisms Underlying the Association Between Frailty and Prognosis

Several mechanisms may explain the association between frailty and poor outcomes. Frailty is defined as an age‐related decline in physiological reserve that increases vulnerability to minor stressors. [6] It has been linked to adverse in‐hospital events such as delirium, falls, and pressure ulcers. [25] The systemic use of corticosteroids in ILD exacerbations may exacerbate frailty‐related vulnerabilities by increasing the risk of delirium through hypothalamic‐pituitary and GABAergic pathway modulation. [26, 27] Delirium itself has been associated with higher in‐hospital mortality. [28] These interacting factors may contribute to the increased mortality observed in frail patients.

Dyspnea, the main perceived symptom of ILD patients, can also be a factor linking frailty and prognosis. Prior studies have stated that the intensity of dyspnea, rather than lung function, is the cause of frailty. [15] In this study, the frail group also had a higher proportion of patients with a worse modified medical research council scale than the nonfrail group. And it has been reported that dyspnea affects prognosis independently of lung function and arterial blood gas values in IPF patients. [29] Although dyspnea was not selected as an independent risk factor for prognosis in our supplemental results, our results suggest that frailty and dyspnea in ILD patients interact with each other to lead to poor outcomes.

Furthermore, functional decline during hospitalization is known to be a risk factor for mortality after discharge in elderly hospitalized patients. [30, 31] Frail patients are more prone to functional decline, and the frail group in this study was largely discharged without full physical recovery, despite receiving standard inpatient pulmonary rehabilitation. Although exercise tolerance, such as 6‐min walking distance, is known to be a predictor of prognosis in ILD patients, [32] our population did not acquire even the more basic ability of activities of daily living. Although the potential mechanisms of functional decline and poor prognosis are unclear, it is a typical clinical course for patients with chronic diseases to experience repeated functional decline due to hospitalization.

### 4.3. Clinical Implications

All hospitalized ILD patients should be assessed for frailty for risk stratification. Although obtaining detailed preadmission information can be challenging, the CFS offers a practical and clinically applicable screening tool. Patients identified as frail may benefit from tailored interventions, including delirium prevention, optimized oxygen therapy to alleviate dyspnea, and intensive rehabilitation strategies such as early ambulation.

## 5. Limitation

First, due to the nature of this study, lung function data prior to hospitalization were often unavailable. As a result, prognostic tools such as the ILD‐GAP score [33] could not be calculated. Nevertheless, multivariate models that included surrogates for disease severity—such as dyspnea, long‐term oxygen therapy, and AE‐ILD history—still identified frailty as an independent prognostic factor. Second, frailty assessments differed across studies. Although we used the CFS for its clinical feasibility, previous reports employed the frailty index or Fried phenotype, limiting direct comparison. It should also be noted that the assessment of frailty prior to hospitalization by CFS may have the potential for recall bias. Third, this study only included Japanese participants, and its wider generalizability is limited. Finally, in‐hospital complications such as delirium, infections, or malnutrition were not systematically evaluated. Future studies should examine how these events mediate the relationship between frailty and prognosis.

## 6. Conclusions

Frailty was independently associated with worse prognosis in ILD patients hospitalized for ARD, even after accounting for treatments during hospitalization and ILD subtype. In addition, patients with frailty were at higher risk of delayed early ambulation. These findings underscore the need for targeted interventions for frail patients in acute care settings and reaffirm the importance of comprehensive frailty management, including pulmonary rehabilitation, in both acute and stable ILD populations.

## Author Contributions

Conceptualization, Y.T. and M.A.; data curation, Y.T. and M.A.; formal analysis, Y.T. and Y.S.; investigation, Y.T.; methodology, Y.T. and M.A.; project administration, Y.T. and M.A.; visualization, Y.T.; writing—original draft, Y.T.; writing—review and editing, Y.T., M.A., T.M., Y.S., S.K., D.M., S.N., and K.Y.; supervision, M.A.

## Funding

No funding was received for this manuscript.

## Disclosure

All authors have read and agreed to the published version of the manuscript.

## Conflicts of Interest

The authors declare no conflicts of interest.

## Supporting information


**Supporting Information** Additional supporting information can be found online in the Supporting Information section. Table S1: Sensitivity analysis for prognostic impact of frailty on 180‐day mortality.

## Data Availability

The datasets used and/or analyzed during the current study are available from the corresponding author on reasonable request.
