# Supplementary material for: Impact of Frailty on Prognosis and Functional Outcome in Patients With Acute Respiratory Deterioration of Interstitial Lung Disease
Source: Pulm Med. 2026 Apr 6;2026:3510542. doi: 10.1155/pm/3510542 (PMC13051896; doi:10.1155/pm/3510542)
Supplement: Supplementary file 1 — Supporting Information Additional supporting information can be found online in the Supporting Information section. Table S1: Sensitivity analysis for prognostic impact of frailty on 180‐day mortality. [file PM-2026-3510542-s001.docx]

Supplemental Table. Sensitivity analysis for prognostic impact of frailty on 180-day mortality

|  | Univariable | |  | Multivariable | |
| --- | --- | --- | --- | --- | --- |
|  | HR (95%CI) | p |  | HR (95%CI) | p |
| Age, 1 year | 1.018 (0.992, 1.045) | 0.178 |  | 1.006 (0.978, 1.035) | 0.681 |
| Male | 1.526 (0.771, 3.020) | 0.226 |  | 1.749 (0.834, 3.665) | 0.139 |
| Steroid pulse | 2.056 (1.152, 3.667) | 0.015 |  | 2.137 (1.179, 3.872) | 0.012 |
| Frailty | 2.404 (1.360, 4.250) | 0.003 |  | 2.474 (1.268, 4.825) | 0.008 |
| Prior LTOT use | 2.082 (1.206, 3.597) | 0.009 |  | 2.261 (1.112, 4.597) | 0.024 |
| Prior ILD exacerbation | 0.995 (0.580, 1.704) | 0.985 |  | 0.590 (0.295, 1.182) | 0.137 |
| Age, 1 year | 1.018 (0.992, 1.045) | 0.178 |  |  |  |
| Mechanical ventilation | 2.328 (0.998, 5.433) | 0.050 |  |  |  |
| UIP/IPF | 1.275 (0.759, 2.144) | 0.359 |  |  |  |
| mMRC scale, 1 grade | 1.273 (1.018, 1.591) | 0.035 |  |  |  |
| Prior ILD diagnosis | 0.908 (0.530, 1.556) | 0.726 |  |  |  |
| Prior ILD exacerbation | 0.995 (0.580, 1.704) | 0.985 |  |  |  |
| AE-ILD | 0.614 (0.365, 1.033) | 0.066 |  |  |  |

*HR* hazard ratio, *CI* confidence interval, *LTOT* long term oxygen therapy, *ILD* interstitial lung diseases, *UIP/IPF* usual interstitial pneumonia / idiopathic pulmonary fibrosis, *mMRC* modified medical research council, *AE-ILD* acute exacerbation of interstitial lung diseases
